# Morphological study of nasal diverticulum in Grant’s zebra (*Equus quagga boehmi* Matschie, 1892)

**DOI:** 10.4142/jvs.25372

**Published:** 2026-07-01

**Authors:** Young-Jin Jang, Dongchoon Ahn

**Affiliations:** 1 Center for Large Animals Convergence Research, Korea Institute of Toxicology, Jeongeup 56212, Korea.; 2 Department of Veterinary Medicine, College of Veterinary Medicine and Institute of Animal Transplantation, Jeonbuk National University, Iksan 54596, Korea.

**Keywords:** Equidae, hair follicle, sebaceous glands, sweat glands, skin

## Abstract

**Importance:**

The nasal diverticulum (ND), a blind cutaneous pouch extending from the nasal vestibule (NV), is unique to Equidae. Although anatomical and histological descriptions of the ND are available for horses and donkeys, detailed studies in zebras remain limited.

**Objective:**

To investigate and characterize the anatomical and histological features of the ND in zebras and compare these findings with those observed in other species.

**Methods:**

Silicone casts of the NDs were prepared from two of three female Grant’s zebra carcasses for anatomical measurements, followed by dissection for gross morphological examination. Three-dimensional reconstructions generated from computed tomography images of the casts were used to obtain morphological measurements. In addition, paraffin-embedded sections stained with hematoxylin and eosin were prepared for histological evaluation and morphometric analysis.

**Results:**

The NDs of zebras were curved blind pouches, slightly compressed mediolaterally, and extended over the nasal process of the incisive bone. Based on the impression of the nasal process, the ND was divided into cranial and caudoventral regions. The nasolacrimal orifice was located on the lateral aspect of the nostril. An undulating fold was observed on the medial wall of the cranial region of the ND. The total volume of the ND was approximately 36 mL. The surface was covered with fine hairs associated with secondary hair follicles. Compared with the NV, the ND exhibited significantly reduced epidermal thickness and fewer sebaceous glands, sweat glands, and secondary hair follicles.

**Conclusions and Relevance:**

This study provides novel anatomical and histological data on the ND in zebras, highlighting species-specific differences compared with other animals.

## INTRODUCTION

The nasal vestibule (NV), the rostral portion of the nasal cavity, is lined with hairy skin and serves as the site of the nasolacrimal duct orifice. The NV is separated from the nasal cavity proper, which is lined by respiratory mucosa, at the limen nasi. Horses and donkeys possess a unique structure, the nasal diverticulum (ND), which extends from the NV [1,2,3,4,5,6,7,8,9,10]. The ND is a cutaneous blind sac extending from the dorsal commissure of the nostril to the caudal end of the nasoincisive notch, and its opening is referred to as the false nostril. It lies on the periosteal membrane and occupies the space between the nasal bone and the nasal process of the incisive bone [1,2,11].

Clinically, the ND is an important structure during nasogastric tube intubation [5,6,7,8]. Recently, it has also been identified as a suitable sampling site for methicillin-resistant *Staphylococcus aureus* infection [12].

Grant’s zebra (*Equus quagga boehmi* Matschie, 1892), the smallest subspecies of the plains zebra in Africa, belongs to the family Equidae, which also includes horses and donkeys [13]. During dissection for veterinary anatomy instruction in our laboratory, we identified the ND in this species. Although we initially expected a structure similar to that of the horse, notable anatomical differences were observed.

Current knowledge of the ND is limited, and only brief descriptions are available for horses and donkeys [1,2,3,9]. In horses, the ND is described as a cone-shaped structure measuring approximately 5–6 cm in length, extending caudally to the angle of junction between the nasal bone and the nasal process of the incisive bone, and lined by finely pigmented skin with sparse fine hairs on the inner surface [1,2,3]. However, Plopper and Adams [14] reported that the inner surface of the equine ND is lined by integument containing vibrissae, sebaceous glands, and sweat glands. In donkeys, the ND has been described as a funnel-shaped pouch divided into cranial and caudal portions [9].

These findings indicate that additional studies are required to elucidate the anatomical and histological characteristics of the ND in equids. However, no information is currently available regarding the ND in zebra for comparison with other equid species. Therefore, the present study was conducted to provide morphometric data and a fundamental anatomical and histological description of the ND in Grant’s zebra.

## METHODS

### Animals

Three female Grant’s zebra carcasses were used in this study: Only female carcasses were available, as the donated animals were determined by the donating institution; no sex-based selection was applied. Sex differences in ND morphology were therefore not assessed. The carcasses were donated by a zoo in Busan, South Korea, in 2021, for educational and research purposes. All the zebras had been housed in the zoo for more than 10 years. Necropsy confirmed no pathological findings in the respiratory tract. The Institutional Animal Care and Use Committee at Jeonbuk National University determined that ethical approval was not required for the use of animal carcasses in this study.

### Silicone casting and gross observation

Silicone casts were prepared to examine the morphology of the ND in two of the three zebras. Commercial silicone (Lucky-Silicone; Wacker Chemical Korea, Korea) was injected into the ND through the nostril and allowed to solidify for 48 h. Following silicone injection, the skin length increased to 110%–115% of its original length (Fig. 1A). A longitudinal incision was then made from the dorsal angle of the nostril caudally along the ND to facilitate careful removal of the cast. After cast retrieval, the external skin of the ND was incised from the dorsal angle of the nostril to the distal end of the pouch (Fig. 1B).

**Fig. 1:**
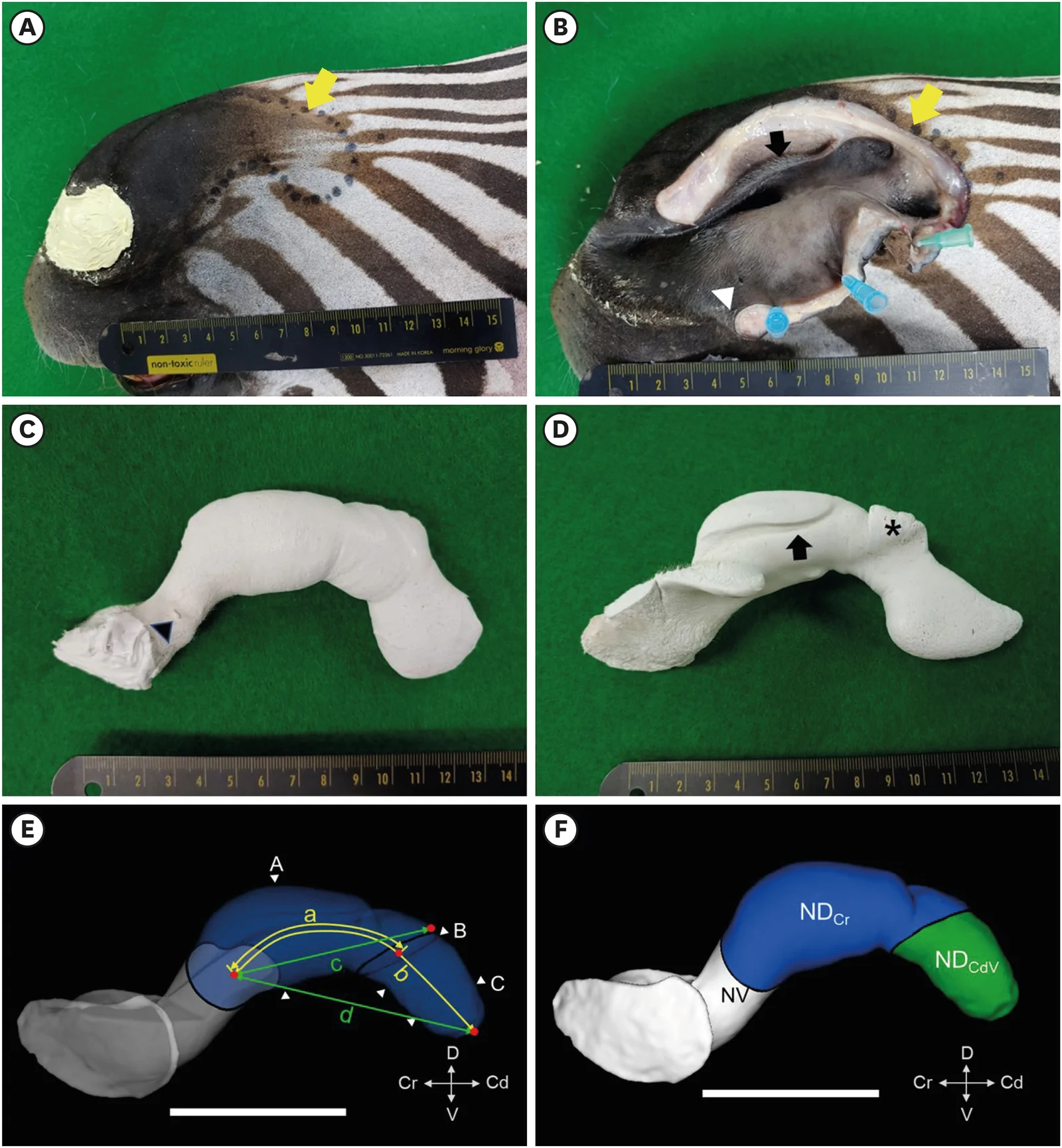
Gross morphology and three-dimensional reconstruction of the ND in the Grant’s zebra. (A) External configuration of the left ND following silicone injection. The swollen margin is indicated by a dotted line. The yellow arrow denotes the angle of the nasoincisive notch. (B) Internal surface of the ND after removal of the silicone cast. The angle of the nasoincisive notch (yellow arrow), the undulating fold (black arrow), and the nasolacrimal orifice (white arrowhead) are visible. (C) Lateral view of the silicone cast of the left ND. The black arrowhead indicates the nasolacrimal orifice. (D) Medial view of the silicone cast of the left ND. The black arrow indicates the impression of the undulating fold. The asterisk denotes a small irregular extension at the caudal end of the cranial portion of the ND. (E) Lateral view illustrating measurement sites for length and width. Length a, curved length of the cranial portion; Length b, total curved length; Length c, straight length of the cranial portion; Length d, total straight length. Plane A, the widest transverse section of the cranial portion; Plane B, the narrowest section at the incisive bone impression; Plane C, the re-expanded section of the caudoventral portion. (F) Lateral view showing the cranial and caudoventral portions of the ND used for volumetric measurements. Scale bar = 5 cm. ND, nasal diverticulum; NV, nasal vestibule; ND_Cr_, cranial portion of the nasal diverticulum; ND_Cdv_, caudoventral portion of the nasal diverticulum; Cr, cranial; Cd, caudal; D, dorsal; V, ventral.

### Computed tomography (CT) scanning and three-dimensional (3D) reconstruction

The silicone casts were scanned using a mobile cone-beam CT system (NFR Phion 1.04; Nanofocusray, Korea) under the following conditions: tube voltage, 105 kV; slice thickness, 0.41 mm; isotropic voxel size, 0.41 mm. Image series were saved in DICOM format. The 3D segmentation and reconstruction of the ND were performed using 3D Slicer (version 5.6.2; Brigham and Women’s Hospital, USA). Volumes, lengths, and transverse breadths were measured using the software’s analytical tools.

### Hematoxylin and eosin (H&E) staining

For tissue sampling, the ventral nasal skin of the NV and ND was divided into three sequential zones from rostral to caudal, resulting in six zones in total (NV1–3 and ND1–3). NV1, NV2, and NV3 corresponded to the rostral, middle, and caudal thirds of the vestibule, respectively. ND1, ND2, and ND3 corresponded to the cranial region, the nasoincisive notch region, and the caudoventral region, respectively. For histological examination, the sampled tissues were fixed in 10% neutral buffered formalin for 48 h, dehydrated, embedded in paraffin, and sectioned at a thickness of 5 µm. Sections were stained with H&E and examined under a light microscope.

For morphometric analysis, Fiji-ImageJ software (version 2.14.0; National Institutes of Health, USA) was used to measure epidermal and dermal thickness and to quantify the density of hair follicles, sebaceous glands, and sweat glands. The size of the cutaneous glands was assessed by measuring their cross-sectional area and the diameters of their long and short axes.

### Statistical analysis

All statistical analyses were performed using Python (version 3.12) and Jamovi (version 2.3.28). Data are expressed as mean ± standard deviation. For numerical variables, differences were assessed using Student’s *t*-test or one-way analysis of variance, followed by Tukey’s *post hoc* test for multiple comparisons. A *p* value < 0.05 was considered statistically significant.

## RESULTS

### Gross observation

Macroscopic examination revealed that the NV was lined with haired skin. A single nasolacrimal orifice was consistently observed in all specimens on the lateral aspect of the nostril. The orifice was located 49.3 mm from the ventral commissure and 11.3 mm from the dorsal commissure within the NV. A marked reduction in hair density was observed caudal to the nasolacrimal orifice, allowing clear differentiation between the NV and the ND. The ND did not terminate at the angle of the nasoincisive notch but extended over the nasal process of the incisive bone and continued onto the maxilla. An undulating fold was observed on the medial wall of the cranial ND. The fold originated independently below the cranial end of the alar fold, extended for approximately 5–6 cm, and terminated at the point where the medial and lateral walls converged. The height of this fold was approximately 1–2 mm. The ND was lined with very fine hairs, which became scarcely visible toward its blind end. The pigmentation of the ND was also markedly reduced compared with the NV (Fig. 1B).

### Measurements of the ND in silicone casting

Examination of the silicone cast revealed that the ND of the zebra was not a simple cone- or funnel-shaped pouch, but an elongated sac with a curved course. It extended caudodorsally within the nasoincisive notch, passed over the nasal process of the incisive bone at the angle of the notch, and then continued caudoventrally to its termination. Accordingly, the ND was defined as having two parts: cranial and caudoventral. The boundary between these parts was marked by an impression left by the nasal process of the incisive bone. Overall, the ND appeared compressed mediolaterally. In the silicone cast, an impression of the undulating fold was also observed in the cranial portion of the ND. In one specimen, a small irregular extension was additionally observed at the angle of the nasoincisive notch (Fig. 1C and D).

CT imaging and 3D reconstruction of the ND revealed that the curved length was 97.5 mm, whereas the straight length was 80.3 mm. The straight length of the cranial part alone was 63.7 mm (Fig. 1E, Supplementary Table 1). The dorsoventral and mediolateral dimensions of the cranial part were 30.3 mm and 21.0 mm, respectively, while those of the caudoventral part were 26.7 mm and 14.2 mm, respectively. The volume of the ND was 24.8 mL for the cranial part and 11.2 mL for the caudoventral part, with a total of 36.0 mL (Fig. 1E and F, Supplementary Table 1).

### Histologic findings

For histological analysis, the NV and ND were each divided into three regions (NV1–3 and ND1–3). Histological examination revealed that the epidermis in both the NV and ND consisted of stratified squamous epithelium with four layers: stratum basale, stratum spinosum, stratum granulosum, and stratum corneum. Dermal papillae were evident at the epidermal–dermal junction. The dermis contained hair follicles, sebaceous glands, and sweat glands; however, arrector pili muscles were absent in both the NV and ND. Neither a cordovan layer nor an accessory cordovan layer, which are present in the general skin of horses and donkeys, was identified. In the NV dermis, distinct bundles of cutaneous muscle were observed between sweat glands, whereas such bundles were absent in the ND (Fig. 2, Supplementary Fig. 1).

**Fig. 2:**
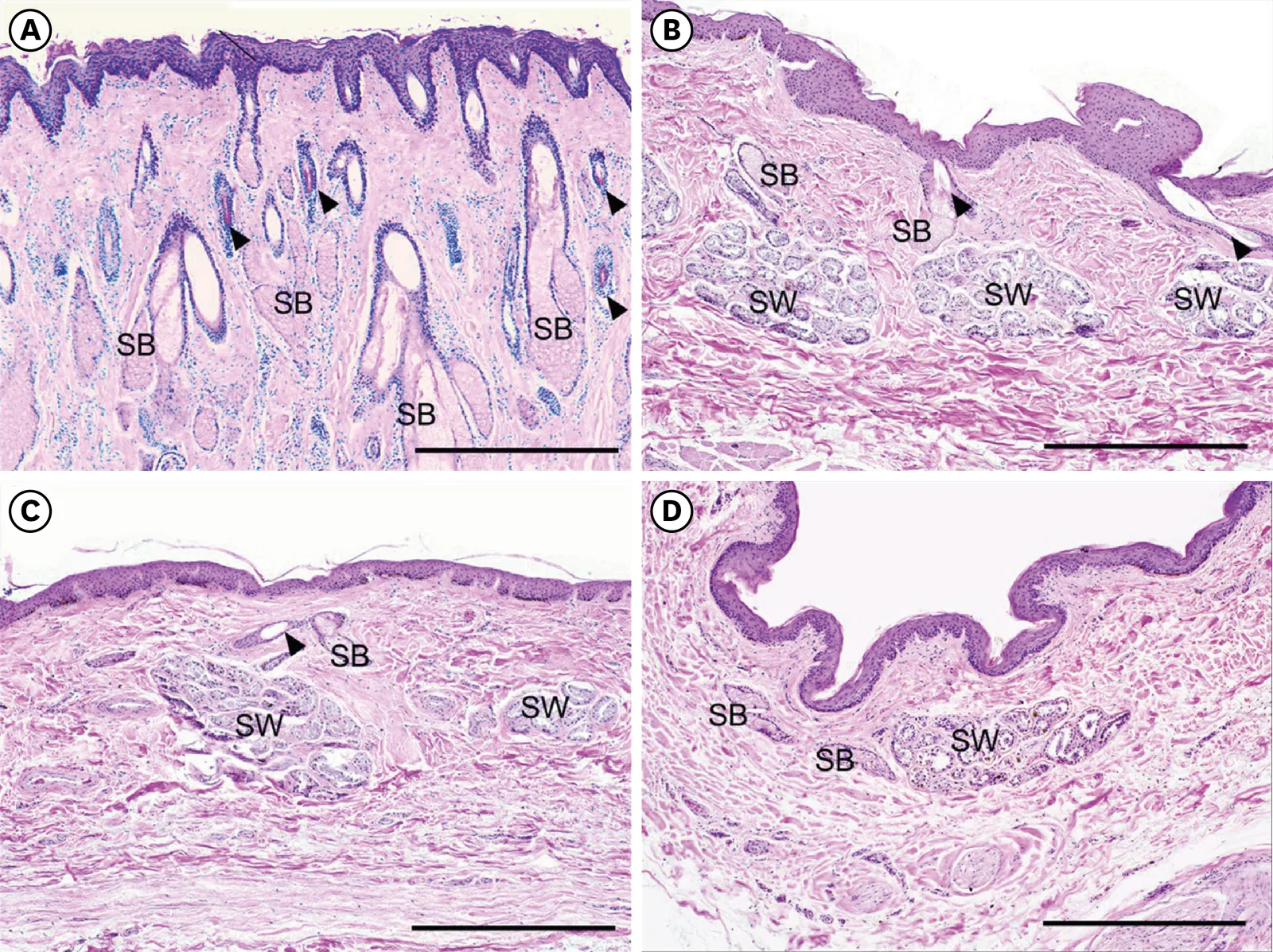
Histological sections of the NV and ND stained with hematoxylin and eosin. (A) Zone 1 of the NV (NV1). (B) Zone 1 of the ND (ND1). (C) Zone 2 of the ND (ND2). (D) Zone 3 of the ND (ND3). Arrowheads indicate hair shafts. Scale bar = 500 μm. NV, nasal vestibule; ND, nasal diverticulum; SB, sebaceous glands; SW, sweat glands.

Epidermal thickness was significantly greater in the NV (62.5 ± 19.7 µm) than in the ND (47.5 ± 14.1 µm; 95% CI, 7.2–22.7 µm; *p* = 0.0002). Dermal thickness was also greater in the NV (2.94 ± 0.66 mm) than in the ND (0.43 ± 0.19 mm; 95% CI, 2.29–2.73 mm; *p* = 1.0 × 10^−35^). Accordingly, the total skin thickness was also greater in the NV (3.01 ± 0.67 mm) than in the ND (0.48 ± 0.19 mm; 95% CI, 2.31–2.75 mm; *p* = 1.2 × 10^−35^). In both the NV and ND, epidermal, dermal, and total skin thickness tended to decrease caudally (Fig. 3, Supplementary Table 2).

**Fig. 3:**
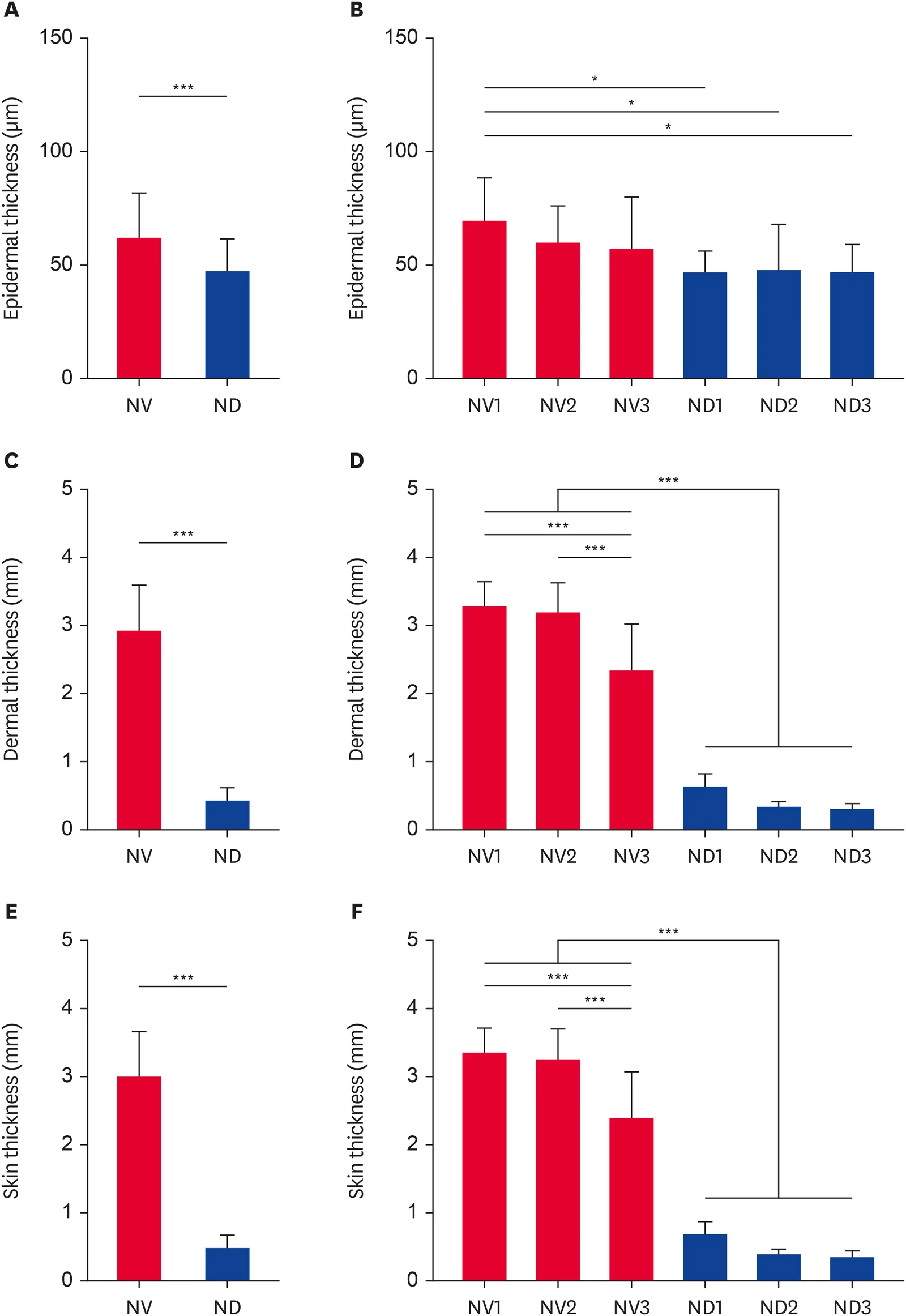
Thickness of the epidermis, dermis, and total skin in the NV and ND. (A) Epidermal thickness in the NV and ND. (B) Epidermal thickness across zones in the NV (NV1–NV3) and ND (ND1–ND3). (C) Dermal thickness in the NV and ND. (D) Dermal thickness across zones in the NV and ND. (E) Total skin thickness in the NV and ND. (F) Total skin thickness across zones in the NV and ND. Statistical analyses were performed using Student’s *t*-test and one-way analysis of variance followed by Tukey’s *post hoc* test. NV, nasal vestibule; ND, nasal diverticulum. ^*^p < 0.05, ^***^p < 0.001. Exact *p* values for all comparisons are given in Supplementary Table 2.

Marked differences were observed in hair follicle density between the NV and ND. Primary hair follicles were present in the NV (1.3 ± 1.1 per mm^2^) but were absent in the ND, and their density decreased markedly in the caudal direction within the NV. Secondary hair follicles were present in both the NV and ND, but their density was significantly higher in the NV (6.0 ± 1.9 per mm^2^) than in the ND (0.3 ± 0.5 per mm^2^; 95% CI, 5.2–6.2 per mm^2^; *p* = 3.9 × 10^−43^) (Fig. 4A-D, Supplementary Table 3).

**Fig. 4:**
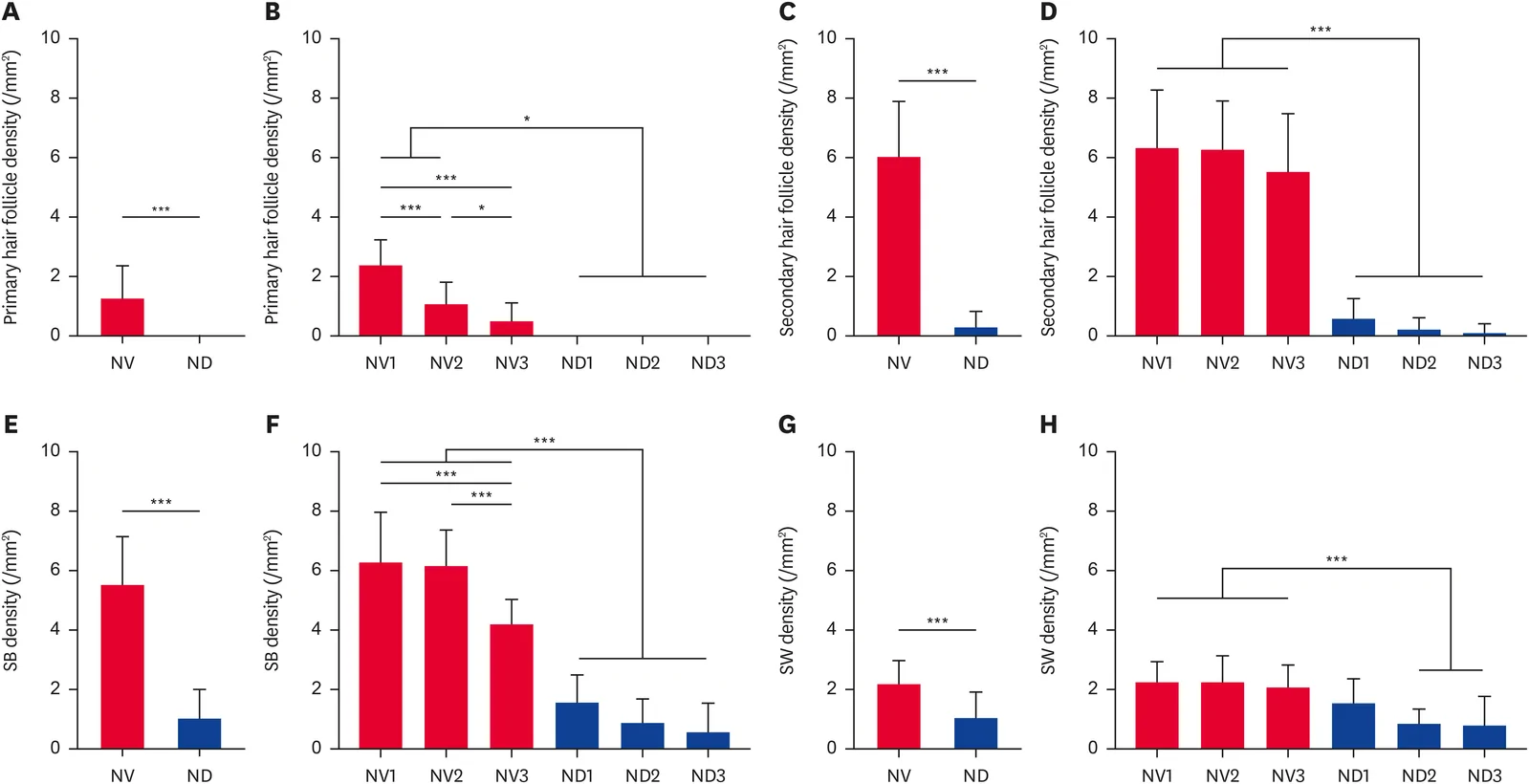
Densities of hair follicles, sebaceous glands, and sweat glands in the NV and ND. (A) Primary hair follicle density in the NV and ND. (B) Primary hair follicle density across zones in the NV (NV1–NV3) and ND (ND1–ND3). (C) Secondary hair follicle density in the NV and ND. (D) Secondary hair follicle density across zones in the NV and ND. (E) Sebaceous gland density in the NV and ND. (F) Sebaceous gland density across zones in the NV and ND. (G) Sweat gland density in the NV and ND. (H) Sweat gland density across zones in the NV and ND. Statistical analyses were performed using Student’s *t*-test and one-way analysis of variance followed by Tukey’s *post hoc* test. NV, nasal vestibule; ND, nasal diverticulum; SB, sebaceous glands; SW, sweat glands. ^*^p < 0.05, ^***^p < 0.001. Exact *p* values for all comparisons are given in Supplementary Table 3.

Sebaceous glands were branched or compound alveolar glands and exhibited holocrine secretion. Sweat glands were simple, coiled tubular glands with apocrine secretion. The density of sebaceous glands was significantly higher in the NV (5.5 ± 1.6 per mm^2^) than in the ND (1.0 ± 1.0 per mm^2^; 95% CI, 4.0–5.0 per mm^2^; *p* = 2.3 × 10^−35^). Similarly, the density of sweat glands was greater in the NV (2.2 ± 0.8 per mm^2^) than in the ND (1.1 ± 0.9 per mm^2^; 95% CI, 0.8–1.4 per mm^2^; *p* = 2.1 × 10^−11^). For both gland types, the density decreased progressively from the NV caudally into the ND (Fig. 4E-H, Supplementary Table 3).

The distribution of sebaceous and sweat glands differed between the NV and ND. Gland distribution was assessed as a depth range, defined as the distance from the epidermal surface to the most superficial and deepest glandular points (minimal to maximal depth). The depth range of sebaceous glands in the NV (440.4 ± 249.6 µm to 740.0 ± 335.6 µm) was deeper than in the ND (158.4 ± 69.6 µm to 265.9 ± 77.1 µm, *p* = 1.6 × 10^−7^ and 3.6 × 10^−10^ for the minimal and maximal depths, respectively). Similarly, the depth range of sweat glands in the NV (2,244.6 ± 750.7 µm to 2,748.9 ± 754.0 µm) was significantly deeper than in the ND (267.7 ± 151.9 µm to 456.1 ± 182.1 µm, *p* = 1.9 × 10^−20^ and 3.5 × 10^−23^ for the minimal and maximal depths, respectively). Across both regions, sebaceous glands were consistently more superficial than sweat glands (*p* = 4.4 × 10^−18^ in the NV and *p* = 0.0007 in the ND) (Fig. 5A-D, Supplementary Table 4).

**Fig. 5:**
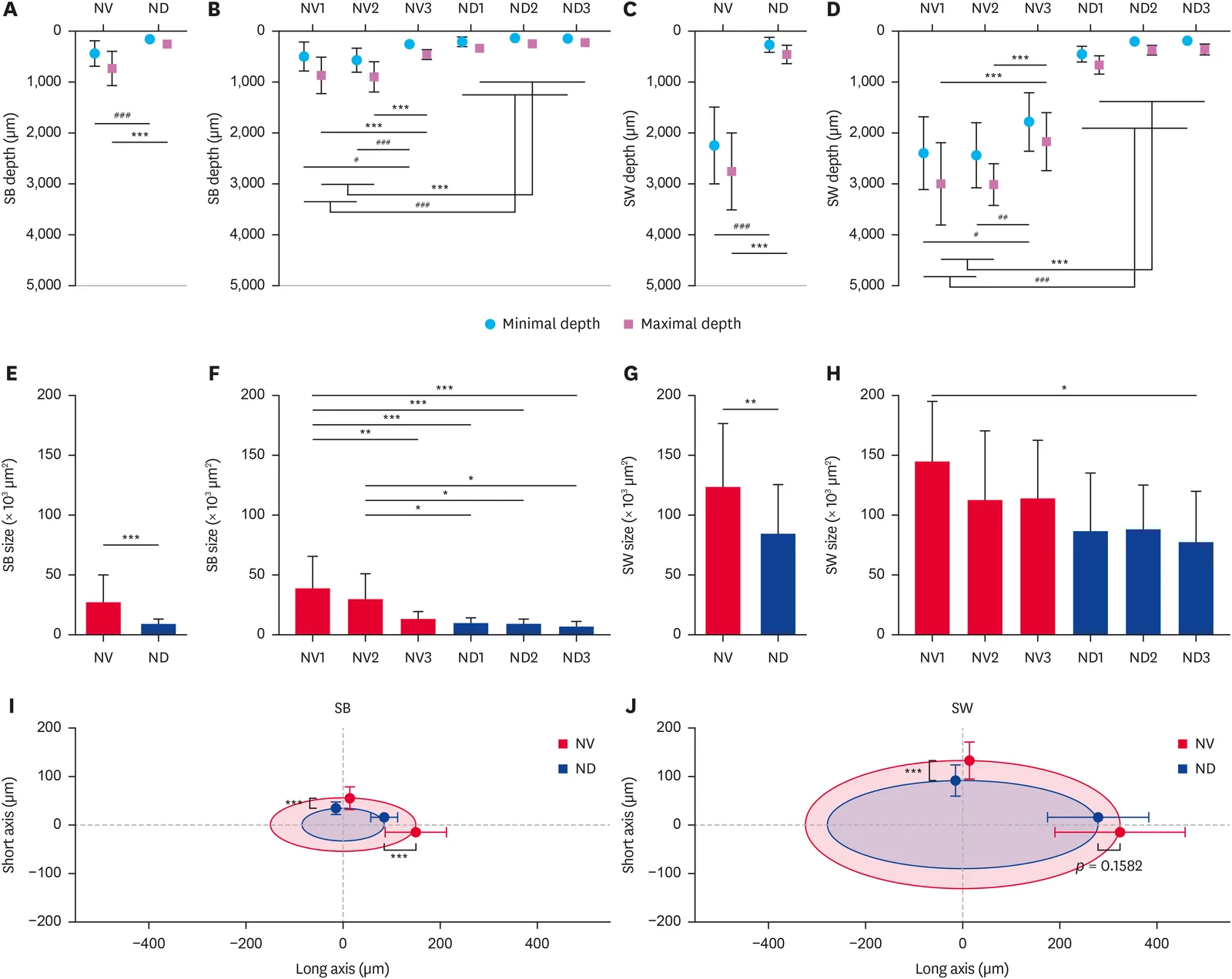
Depth distribution and size of SB and SW in the NV and ND. (A, B) Minimal and maximal depth ranges of sebaceous glands in the NV and ND (A) and within each zone (NV1–3 and ND1–3) (B). (C, D) Depth ranges of sweat glands in the NV and ND (C) and within each zone (D). (E, F) Cross-sectional area of sebaceous glands in the NV and ND (E) and within each zone (F). (G, H) Cross-sectional area of sweat glands in the NV and ND (G) and within each zone (H). (I, J) Elliptical plots showing long- and short-axis diameters of sebaceous glands (I) and sweat glands (J) in the NV and ND. Statistical analyses were performed using Student’s *t*-test and one-way analysis of variance with Tukey’s *post hoc* test. SB, sebaceous glands; SW, sweat glands; NV, nasal vestibule; ND, nasal diverticulum. ^*^p < 0.05, ^**^p < 0.01, ^***^p < 0.001 for maximal depth or size comparisons; ^#^*p* < 0.05, ^##^*p* < 0.01, ^###^*p* < 0.001 for minimal depth comparisons. Exact *p* values for all comparisons are given in Supplementary Tables 4 and 5.

The size of the sebaceous and sweat glands also differed between the NV and ND. Gland size was evaluated as the cross-sectional area on histologic sections. Sebaceous glands were larger in the NV (27,100 ± 22,400 µm^2^) than in the ND (8,500 ± 4,400 µm^2^, *p* = 4.1 × 10^−5^). Sweat glands were also larger in the NV (123,600 ± 52,600 µm^2^) than in the ND (83,900 ± 41,600 µm^2^, *p* = 0.0020). For sebaceous glands, both the long and short axis diameters were greater in the NV (302.1 ± 125.7 µm and 108.9 ± 48.3 µm, respectively) than in the ND (170.4 ± 55.7 µm and 67.5 ± 26.0 µm, *p* = 2.3 × 10^−6^ and *p* = 0.0001, respectively). For sweat glands, the short axis diameter was greater in the NV (264.6 ± 76.7 µm) than in the ND (183.3 ± 63.0 µm, *p* = 3.4 × 10^−5^), whereas the long axis did not differ significantly between the NV (648.5 ± 268.9 µm) and the ND (559.8 ± 207.8 µm, *p* = 0.1582) (Fig. 5E-J, Supplementary Table 5).

Melanin pigments were observed in the epidermis, hair follicles, sebaceous glands, and sweat glands. In the epidermis of NV1 and NV2, melanin granules were present in the stratum basale, stratum spinosum, and stratum granulosum. In the latter two layers, granules were aggregated on the apical side of the keratinocyte nuclei, forming apical caps. In contrast, in the epidermis of NV3 and ND1–ND3, melanin granules were confined to the stratum basale, and their frequency decreased progressively toward ND3. The melanin granules in hair follicles, sebaceous glands, and sweat glands did not appear to differ across the NV and ND regions. An undulating fold was observed in the cranial part of the ND, the center of which consisted primarily of fibrous connective tissue.

## DISCUSSION

This study provides the first comprehensive description of the ND in zebras. To date, a pouch or recess associated with the NV has been described only in horses [1,2,3,4,5,6,7], donkeys [8,9,10], camels [9,15,16], moose [17], and the saiga antelope [18,19]. In horses and donkeys, the ND is clinically relevant, as it requires consideration during intubation to prevent misplacement of nasogastric tubes [3,5,6,7,8]. It also serves as a site for microbial sampling [12]. In camels, a lateral ND has been described [9,15,16], while in the saiga antelopes, a lateral vestibular recess has been reported [18,19]. These structures have been hypothesized to facilitate the generation of negative pressure during nostril closure, thereby contributing to respiratory regulation. They may also humidify inspired air, protect the nasal mucosa, and enhance olfactory detection of odorant substances [15,16,17]. Accordingly, characterization of the ND across species is important not only for comparative anatomy but also for understanding species-specific adaptations of the nasal cavity.

The zebra ND more closely resembles that of the donkey than that of the horse, as it extended beyond the incisive bone. In horses, the ND is typically described as a simple cone-shaped pouch [1,2,3]. In donkeys, however, available descriptions are limited and inconsistent. Abdallah et al. [10] characterized the ND as a simple conical pouch, whereas Metwally et al. [9] described a funnel-shaped structure comprising cranial and caudal parts. According to Metwally et al. [9], the cranial part is located in the nasoincisive notch, while the caudal part extends laterally to the nasal process of the incisive bone and the maxilla. A further distinction is that, in donkeys, the undulating fold lies on the lateral wall and extends toward the alar fold, whereas in zebras it is located on the medial wall (Fig. 6) [9].

**Fig. 6:**
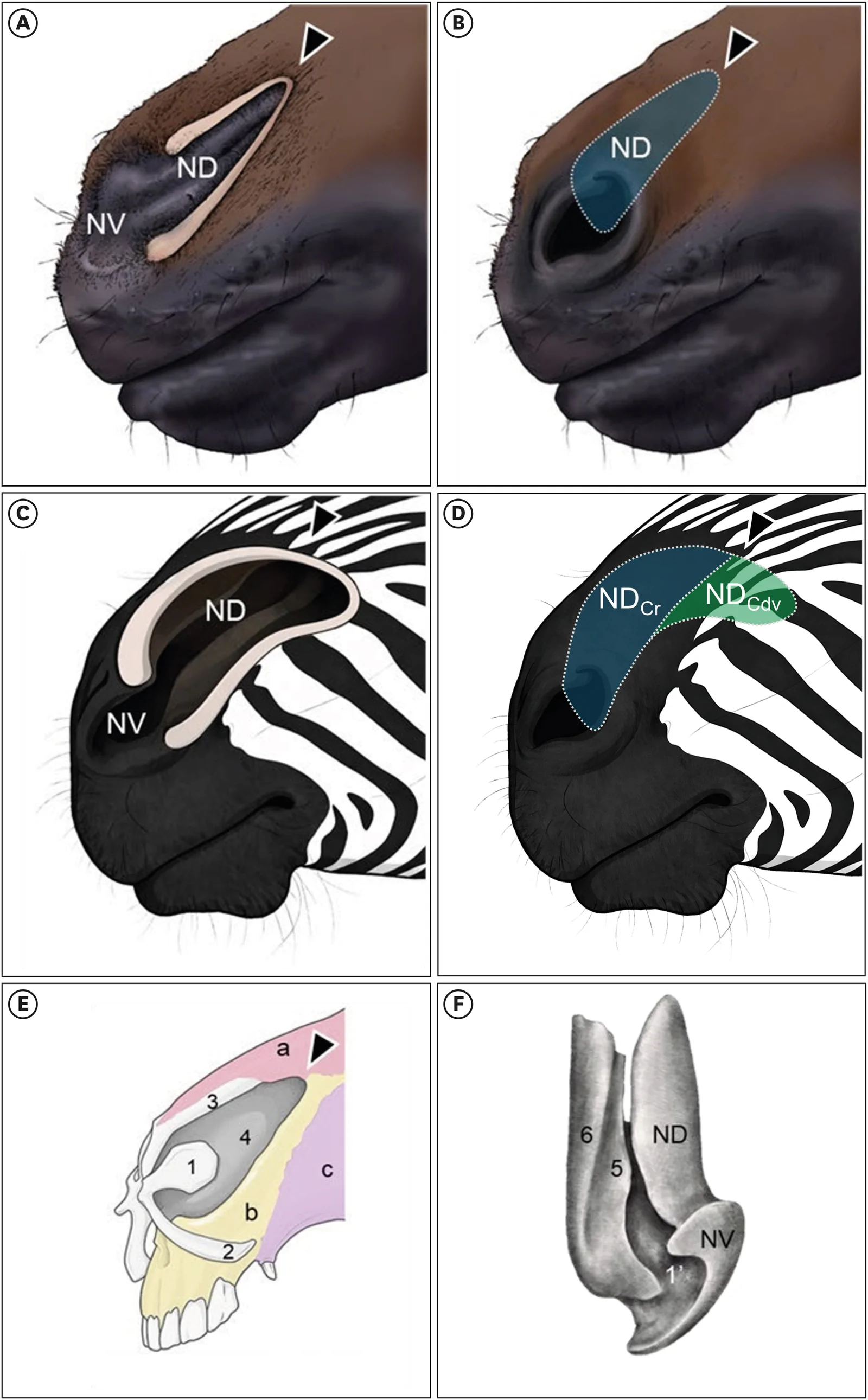
Comparative schematic illustrations of the ND in the horse and the Grant’s zebra. (A) Left nostril of the horse after skin incision, showing the ND and NV. (B) External view of the horse nostril prior to incision, illustrating the projected location of the ND. (C) Left nostril of the Grant’s zebra after skin incision, showing the ND and NV. (D) External view of Grant’s zebra nostril prior to incision, illustrating the projected locations of the ND_Cr_ and ND_Cdv_. (E) Nasal cartilages and adjacent bones associated with the horse ND: a, nasal bone; b, incisive bone; c, maxilla; 1, lamina of the alar cartilage; 2, cornu of the alar cartilage; 3, dorsal lateral nasal cartilage; 4, nasal septum. (F) Dorsal view of a cast of the left nostril, ND, and NV of the horse. 1’, space corresponding to the alar fold; 5, groove corresponding to the dorsal turbinate fold; 6, dorsal meatus. (A), (B), and (E) were modified from *Dyce, Sack, and Wensing’s Textbook of Veterinary Anatomy* [7], and (F) was modified from *Sisson and Grossman’s Anatomy of the Domestic Animals* [3]. Arrowheads indicate the nasoincisive notch. ND, nasal diverticulum; NV, nasal vestibule; ND_Cr_, cranial portion of the nasal diverticulum; ND_Cdv_, caudoventral portion of the nasal diverticulum.

These anatomical differences were also reflected in the size of the ND. The straight length of the zebra ND (80.3 mm) substantially exceeds that reported in horses (50–60 mm) [1,2,3]. Given that the cranial part alone measured 63.7 mm, the additional length is attributable to the caudoventral part extending over the nasal process of the incisive bone. Because Grant’s zebra (approximately 233 kg) is smaller than horses (approximately 500 kg), the ND appears disproportionately large relative to body mass [20]. This difference may reflect species-specific adaptations shaped by the ecological demands of the East African savanna habitat [21,22,23].

Our study provides new histological findings about hair follicles in the NV and ND of zebras. In the NV, both primary and secondary follicle types were present, whereas the ND contained only sparse secondary follicles and no primary follicles. Accordingly, the hair in the zebra ND can be classified as fine hairs. This finding contrasts with a previous report that described the presence of vibrissae in the ND [14], but is consistent with another study reporting fine hairs in regions such as the auricles, nares, lips, anal region, teats, and the ventral tail [24].

Hair follicle density in the zebra NV and ND also differed from previous observations in equids. In the present study, the zebra NV exhibited mean densities of 1.3 follicles/mm^2^ for primary follicles and 6.0 follicles/mm^2^ for secondary follicles, whereas the ND showed only sparse secondary follicles (0.3 follicles/mm^2^). In Italian horses, primary follicle density ranges from 1.6 to 3.8 follicles/mm^2^ and secondary follicle density from 1.0 to 2.5 follicles/mm^2^ [25]. In Arabian horses, seasonal variation has been reported, with densities ranging from 1.5 to 3.8 follicles/mm^2^ in winter and 1.0 to 3.1 follicles/mm^2^ in summer [26,27]. Donkeys exhibit substantially higher densities, ranging from 5.6 to 16.3 follicles/mm^2^ [28]. Collectively, these findings suggest that hair follicle density in the zebra NV is lower than in donkeys but slightly higher than in horses. In contrast, the ND shows markedly low follicle density, consistent with an almost hairless structure.

A further notable finding was the absence of arrector pili muscles from both primary and secondary follicles in the NV and ND of zebras. This suggests that hairs in these regions do not contribute to thermoregulatory functions such as piloerection. Arrector pili muscles are generally absent in areas where hair is sparse or of limited functional importance [24]. Given the adaptation of zebras to hot environments, the absence of these muscles in the NV and ND is likely functionally appropriate.

Both sebaceous and sweat glands were identified in the NV and ND of zebras. However, glandular density differed markedly between these regions, likely reflecting differences in the frequency and intensity of external stimulation. Cutaneous glandular structures are known to diminish in size and number in regions subject to minimal mechanical or environmental stimuli [29]. The lower glandular density observed in the ND is therefore likely attributable to its more protected anatomical position and reduced exposure to external stimuli compared with the NV.

In donkeys, sebaceous gland density has been reported to range from 3.0 to 7.0 glands/mm^2^ across body regions, whereas sweat gland density is considerably higher, ranging from 19.3 to 33.6 glands/mm^2^ [28]. In comparison, sebaceous gland density in the zebra NV was similar to that of donkeys, but markedly lower in the ND. Sweat gland density, however, was substantially lower in zebras than in donkeys, suggesting species-specific differences in cutaneous gland distribution.

Similar to gland density, the size of sebaceous and sweat glands in zebras decreased progressively from the NV to the ND. To date, the only available measurements of gland size in equids have been reported by Sathapathy et al. [30] in zebras. They described sebaceous glands as varying by body site, with long and short axis diameters ranging from 69.6–198.2 μm and 23.3–91.7 μm, respectively. In comparison, sebaceous glands in the zebra NV were larger than those reported for general body regions, whereas those in the ND were of comparable size.

Previous studies have demonstrated that skin thickness—defined as the combined thickness of the epidermis and dermis—varies considerably among individuals, breeds, and anatomical regions in equids [24,31,32,33]. In horses, epidermal thickness has been reported to range from 15–80 μm [34,35,36], with certain regions such as the upper and lower lips and nostrils exceeding 200 μm [24]. Total skin thickness averages approximately 3.8 mm but shows substantial regional variation, ranging from 1.2 to 7.0 mm [24,37]. In donkeys, epidermal thickness typically ranges from 30–100 μm, while dermal thickness ranges from 2.5–3.5 mm [28].

In the present study, the zebra NV exhibited an epidermal thickness of approximately 62.5 μm and a dermal thickness of 2.94 mm, yielding a total thickness of about 3.01 mm. In contrast, the ND showed markedly reduced values, with an epidermal thickness of 47.5 μm, a dermal thickness of 0.43 mm, and a total thickness of only 0.48 mm. Compared with previous reports, the total skin thickness of the zebra NV falls within the range described for horses and donkeys, whereas that of the ND represents the thinnest skin structure documented to date in equids. This pronounced reduction in thickness may be attributable to its internal location, which is less exposed to external mechanical and environmental stimuli, as well as to the absence of a cordovan layer [24,37].

In horses and donkeys, the dermis contains a distinct dense collagenous layer known as the cordovan layer (or third layer), located deep to the reticular dermis [24,28,35,37,38]. Accessory cordovan layers have also been reported in regions such as the lumbar, sacral, gluteal, femoral, metacarpal, digital, and metatarsal areas [37,38]. In contrast, neither a cordovan layer nor accessory cordovan layers were observed in the zebra NV or ND. Accordingly, the deepest dermal structures identified in these regions were sweat glands within the reticular dermis. It remains unclear whether the cordovan layer is entirely absent throughout zebra skin; however, its absence was clearly confirmed in both the NV and ND.

In horses, melanin granules are known to extend through the stratum spinosum and stratum granulosum of the epidermis [24]. In the present study, melanin pigmentation in zebras was observed up to the stratum granulosum in NV1 and NV2, whereas from NV3 to ND3, it was restricted to the basal layer. This variation is likely attributable to differential exposure to sunlight, as NV1 and NV2 are directly exposed when the nostril is open, whereas the remaining regions are comparatively shielded. In addition, melanin granules were also identified in hair follicles, sebaceous glands, and sweat glands, consistent with previous observations in horses [14].

This study has several limitations. First, the small number of animals examined limits the generalizability of the findings. Second, all specimens were female, so potential sex effects could not be assessed. Third, the animals had been housed in a zoo for more than 10 years, and the possible effects of prolonged captivity and age on skin thickness, glandular density, and coat characteristics warrant caution in relating the present findings to savanna habitat adaptation. Fourth, multiple histological measurements were obtained from a limited number of animals, which should be considered when interpreting the quantitative findings. Finally, comparable quantitative reference data for zebra integument are limited.

In conclusion, this study provides a detailed gross anatomical and histological characterization of the ND in zebras. These findings establish baseline morphological data and provide a useful reference for future comparative studies across equids and other species.

## SUPPLEMENTARY MATERIALS

Supplementary Table 1 Morphometric measurements of the ND in Grant’s zebra based on three-dimensional reconstructed images

Supplementary Table 2 The thicknesses of the epidermis, dermis, and skin in different zones of the NV and ND in Grant’s zebra

Supplementary Table 3 The densities of hair follicles, sebaceous glands, and sweat glands in different zones of the NV and ND in Grant’s zebra

Supplementary Table 4 The depth ranges of sebaceous glands and sweat glands from the epidermal surface in different zones of the NV and ND in Grant’s zebra

Supplementary Table 5 The sizes and diameters of sebaceous glands and sweat glands in different zones of the NV and ND in Grant’s zebra

Supplementary Fig. 1 Representative histological images of the NV. (A) NV1, rostral third. (B) NV2, middle third. (C) NV3, caudal third. Scale bar = 500 μm.
